# Comparison of GPT-5 Responses With the Official Results of the Polish Specialized Psychiatric Examination in Child and Adolescent Psychiatry

**DOI:** 10.7759/cureus.92982

**Published:** 2025-09-22

**Authors:** Anna Kowalczyk, Michalina Loson-Kawalec, Aleksander Tabor, Patrycja Dadynska, Katarzyna Romanowicz, Aleksandra Wielochowska, Dawid Boczkowski, Weronika Majchrowicz, Tomasz Dolata, Piotr Sawina, Dawid Bartosik, Alicja Szalach, Marta Zerek, Gracjan Sitarek, Dominika Radej

**Affiliations:** 1 Faculty of Medicine, University of Opole, Opole, POL; 2 Department of Internal Medicine, Karol Marcinkowski University Hospital, Zielona Góra, POL; 3 Department of Internal Medicine, Central Teaching Hospital of the Medical University of Lodz, Lodz, POL; 4 Department of Internal Medicine, Multispecialty Independent Public Health Care Institution Hospital in Nowa Sól, Nowa Sól, POL; 5 Department of Internal Medicine, Non-public Health Care Institution (NZOZ) Hospital in Dzierżoniów, Dzierżoniów, POL; 6 Department of General and Vascular Surgery, Specialist Medical Center, Polanica-Zdrój, POL

**Keywords:** artificial intelligence in education, chat gpt-5, child and adolescent psychiatry, medical education technology, pes

## Abstract

Introduction

Artificial intelligence (AI), particularly language models such as ChatGPT (OpenAI, San Francisco, CA, USA), is becoming increasingly important in medical education and knowledge assessment. Prior studies have demonstrated the growing effectiveness of AI in preparing students for medical examinations, including the Medical Final Examination (Lekarski Egzamin Końcowy (LEK)) of Poland and the National Specialty Examination across various disciplines. This raises important questions regarding its potential role as a tool to support specialist training.

Objective

The aim of this study is to evaluate the effectiveness of the advanced GPT-5 model in addressing problems in child and adolescent psychiatry. The focus is on the accuracy of answers, their correctness, and the model’s self-declared confidence levels to assess its potential efficacy in education.

Methodology

The study analyzed the official spring 2025 National Specialty Examination (Państwowy Egzamin Specjalizacyjny (PES)) of Poland in child and adolescent psychiatry. The exam consisted of 120 multiple-choice questions with a single correct answer. GPT-5 was familiarized with the examination rules and then presented with the questions in the Polish language. Answers were evaluated using the official Centre for Medical Examination (CEM) key. In addition, the model provided a confidence rating for each answer on a five-point scale. Questions were categorized as either clinical or theoretical. Statistical analysis was conducted using the chi-square test and the Mann-Whitney U test.

Results

GPT-5 answered 97 questions correctly (80.8%), surpassing the required passing threshold. No significant difference was observed between the accuracy of responses to clinical versus theoretical questions (p = 0.399). However, correct answers were significantly more likely when the model reported higher confidence levels (p = 0.012).

Conclusions

GPT-5 demonstrated strong performance in the National Specialty Examination of Poland in child and adolescent psychiatry, supporting its potential as a supplementary tool in specialist education. Confidence ratings may provide an additional metric for evaluating the reliability of answers. Nevertheless, broader integration of AI in medical education requires experts overseeing the process and further research across diverse medical disciplines.

## Introduction

ChatGPT, also known as Chat Generative Pre-trained Transformer, is a conversational program or chatbot based on a large language model (LLM). This type of artificial intelligence (AI) was created by OpenAI, San Francisco, CA, USA, in 2022 [1]. GPT-5 is the latest version of the large language model developed by OpenAI, released on August 7, 2025. It was designed based on the GPT architecture while incorporating innovations from thought-based models such as o1 and o3 [2].

In recent years, AI has been becoming increasingly influential in healthcare, with applications ranging from mortality risk prediction in intensive care units to drug discovery and the evaluation of surgical outcomes. The rapid growth of interest in AI is reflected in the rising number of stakeholders in this field, with OpenAI among the leading corporations.

The GPT series, particularly ChatGPT, has established new benchmarks in natural language processing by applying machine learning techniques to analyze and generate language with human-like nuance and complexity [3]. According to Semrush data (July 2025), ChatGPT ranks as the fifth most visited website globally, while the OpenAI website ranks 29^th^, with over one billion monthly visits. In February 2025, OpenAI’s Chief Operating Officer Brad Lightcap reported 400 million weekly active users, a figure that increased to 800 million by April, as documented in Mary Meeker’s AI report. In July 2025, CEO Sam Altman confirmed that users were generating 2.5 billion daily messages [4].

Generative AI, including language models such as ChatGPT, is increasingly playing a role in child and adolescent psychiatry. It can help clinicians screen for and detect disorders like autism, mood disorders, and anxiety, as well as monitor symptoms in teenagers. This allows for earlier intervention than traditional clinical methods [5]. National Institute for Health and Care Excellence (NICE) guidelines for diagnosing autism in individuals under 19 emphasize the use of standardized assessment tools, timely referral to specialists, and support for families. These guidelines provide a useful framework for integrating digital tools and AI into everyday clinical practice [6]. Moreover, AI-based predictive models can identify risk factors for mental health disorders in adolescents before clear symptoms appear, opening the door to preventive interventions [7].

Currently, the adoption of ChatGPT in medicine is rapidly expanding in the United States. A U.S. study aimed at showcasing the program’s ability to take part in advanced-level medical tests and examinations showed that ChatGPT successfully passed the U.S. Medical Licensing Examination (USMLE) with a score of 60% [8]. In contrast, the tool has not yet been adapted for Polish clinical documentation, medical education, or data collection. A recent study by Kufel et al. evaluated ChatGPT’s performance on the Polish National Specialty Exam in radiology. Although the model did not pass, in some subcategories it approached the required threshold. National specialization examinations vary in difficulty across disciplines, but they are generally more demanding than the Medical Final Examination (Lekarski Egzamin Końcowy (LEK)) of Poland.

Considering the surrounding circumstances, this study investigates the performance of GPT-5 in the National Specialty Exam (Państwowy Egzamin Specjalizacyjny (PES)) of Poland in child and adolescent psychiatry, with a focus on its strengths and limitations in comparison with human reasoning.

## Materials and methods

The study was conducted between August 11 and 15, 2025, using the GPT-5 model. The analyzed exam was the 2025 spring PES in child and adolescent psychiatry, randomly selected from the Centre for Medical Examination (CEM) archive in Łódź, Poland. The questions and official answers are publicly available and widely accessible. It contained 120 multiple-choice questions, each with five options and one correct answer. The Examination Committee confirmed that all questions were answered appropriately to current knowledge criteria.

Questions were classified into two categories: clinical cases, requiring analysis of symptoms, diagnostic findings, and treatment decisions; and other topics, including theoretical knowledge, standards of care, and general information unrelated to specific patient cases. Classification was independently performed by two researchers, with any discrepancies appearing resolved through consensus with a third expert.

Answers were scored based on the official key provided by CEM. All questions and answers were carefully recorded. After each question was entered into the model, ChatGPT was asked to rate its level of confidence in the answer, asking, "On a scale of one to five, how confident are you in your answer to this question?" The purpose of these questions was to assess ChatGPT's confidence in answering. The model could assign one of the following ratings: 1: no confidence, 2: low confidence, 3: moderate confidence, 4: high confidence, and 5: complete confidence.

Every question was input into the chat, and all interactions were documented. The exchange took place entirely in the Polish language to ensure cohesion with the Polish specialty examination in child and adolescent psychiatry.

The GPT-5 model used in this study was pre-trained and did not have access to the internet or any external databases while answering the questions. All responses were generated solely based on the model’s internal knowledge, without live web searches or real-time information retrieval.

Statistical analysis was performed with the use of the Microsoft Excel (Microsoft® Corp., Redmond, WA, USA) program and GraphPad Prism 10 (GraphPad Software, La Jolla, CA, USA). The chi-square test compared the number of correct vs. incorrect responses across categories of clinical questions and others. The Mann-Whitney U test evaluated confidence levels between correct and incorrect responses. A p-value < 0.05 was considered statistically significant.

Collaboration among research centers was conducted via online tools, including Microsoft Teams (Microsoft® Corp., Redmond, WA, USA), Facebook Messenger (Meta Platforms, Inc., Menlo Park, CA, USA), e-mail, and Google Docs (Google, Inc., Mountain View, CA, USA). All sections of the manuscript created by different research teams were peer reviewed with the use of the aforementioned tools to ensure consistent contributions to this study.

This study did not involve human participants or private patient data, and all exam materials are publicly available. Therefore, formal ethical approval was not required. The study was conducted following principles of integrity, transparency, and good research practice.

## Results

GPT-5 correctly answered 97 of 120 questions (80.8%), with 23 incorrect responses (19.2%) (Table 1). Appendix A includes questions from the PES examination in child and adolescent psychiatry, spring 2025, translated into English, along with the answers provided by GPT-5 and its level of confidence in the correctness of these answers. No significant difference was observed between performance on clinical versus non-clinical questions (p = 0.399, χ² = 0.713) (Table 2). Confidence ratings were significantly higher for correct answers compared with incorrect ones, suggesting the confidence levels could be used as an answer accuracy measurement (U = 944; p < 0.05).

**Table 1:** Comparison of the responses of CEM and GPT-5 to questions from the Child and Adolescent Psychiatry Specialist Examination with declared confidence levels. The table illustrates the model's responses in terms of correct answers, according to the official answer key provided by the Medical Examination Center in Łódz. Additionally, for each question, the model's confidence level (1-5). CEM: Centre for Medical Examination

Question number	Answers: CEM	Answers: GPT-5	Confidence: GPT-5 (1-5)
1	A	A	5
2	D	D	5
3	D	D	5
4	A	A	5
5	B	B	5
6	D	D	5
7	C	C	5
8	D	D	5
9	A	A	5
10	C	C	5
11	E	E	5
12	C	C	5
13	D	D	5
14	A	A	5
15	D	E	5
16	B	B	5
17	A	A	5
18	E	E	5
19	D	C	5
20	A	A	5
21	B	B	5
22	E	E	5
23	C	C	5
24	A	D	5
25	B	B	5
26	E	E	5
27	C	D	5
28	C	C	5
29	E	E	5
30	B	B	5
31	D	D	5
32	A	D	5
33	C	D	5
34	D	D	5
35	B	B	5
36	E	E	5
37	B	B	5
38	A	A	5
39	A	C	5
40	D	D	5
41	B	B	5
42	E	E	5
43	B	B	5
44	D	D	5
45	D	D	5
46	D	C	5
47	E	E	5
48	B	A	4
49	E	E	5
50	B	B	5
51	A	A	5
52	D	E	5
53	C	C	5
54	D	D	5
55	D	D	4
56	C	C	5
57	C	C	5
58	D	B	5
59	E	E	5
60	C	A	5
61	A	A	5
62	A	B	5
63	E	C	5
64	B	B	5
65	C	C	5
66	B	B	5
67	D	D	5
68	E	E	5
69	C	C	5
70	E	E	5
71	B	B	5
72	A	B	4
73	D	D	5
74	B	B	5
75	E	E	5
76	A	A	5
77	E	E	5
78	D	D	5
79	E	D	5
80	A	A	5
81	E	E	5
82	A	A	4
83	E	E	5
84	B	B	5
85	B	B	5
86	A	A	5
87	B	B	5
88	C	C	5
89	D	D	5
90	B	B	5
91	B	B	5
92	A	C	5
93	E	E	5
94	A	A	5
95	E	E	5
96	C	C	5
97	C	C	5
98	B	B	5
99	D	D	5
100	B	A	4
101	E	E	5
102	C	C	5
103	C	C	5
104	D	A	4
105	D	D	5
106	E	C	5
107	C	C	5
108	A	A	5
109	D	D	5
110	B	A	5
111	B	A	5
112	A	A	5
113	D	C	5
114	E	E	5
115	C	C	5
116	A	A	5
117	D	C	5
118	B	B	5
119	C	C	5
120	E	E	5

**Table 2:** GPT-5's effectiveness by query type: clinical vs. theoretical situations Results are expressed as numbers (N) and percentages (%). Statistical analysis revealed no significant differences between the groups (p = 0.399; χ² = 0.713).

Category	(N) Correct	(N) Incorrect	% Correct	% Incorrect	p-Value χ² Value
Clinical	25	4	86.2%	13.8%	p-Value = 0.399 χ²-Value= 0.713
Theoretical (Others)	72	19	79.1%	20.9%	

**Figure 1:**
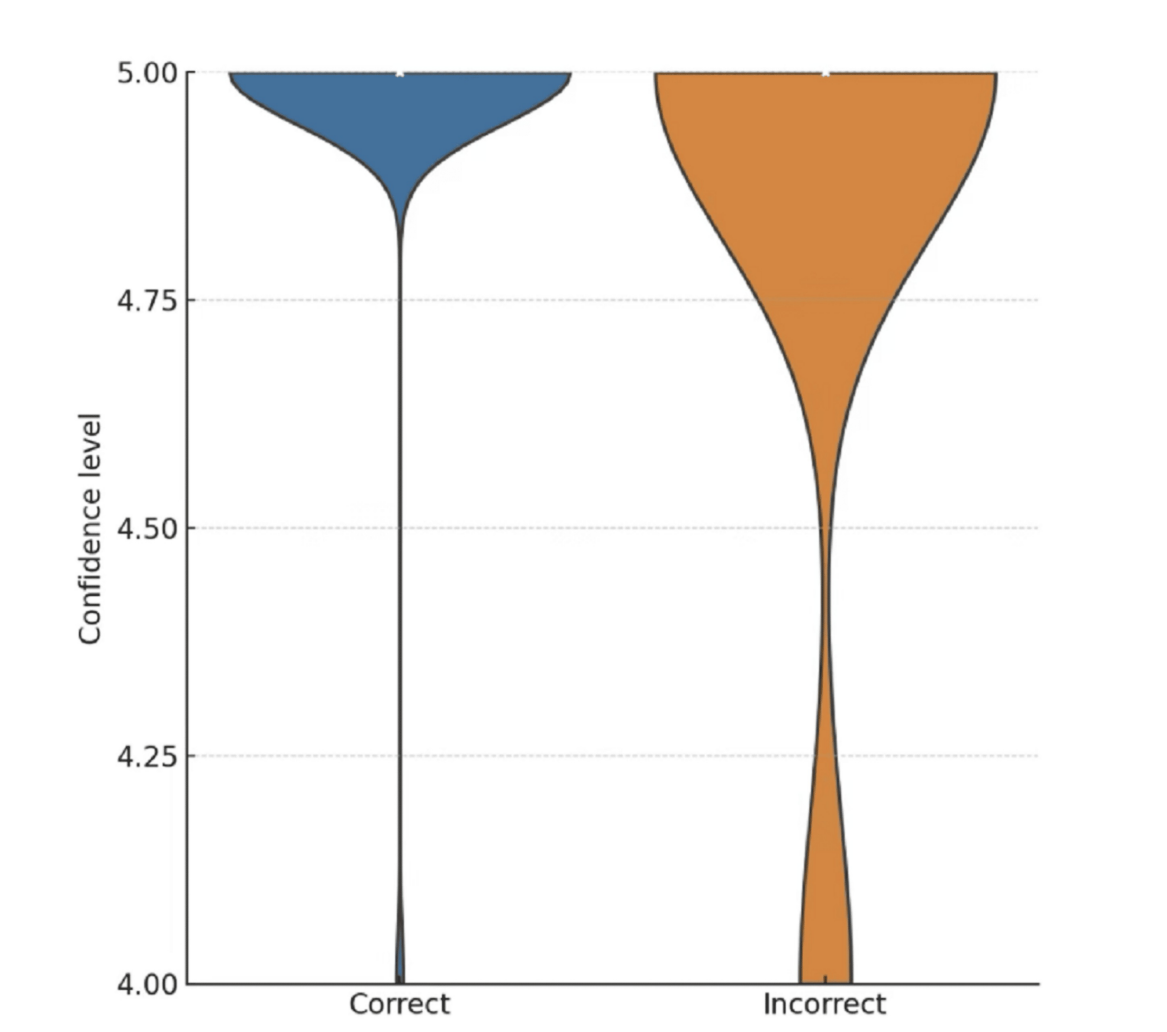
Reported confidence level of the GPT-5 model in terms of response accuracy The data are shown separately for correct and incorrect responses. The confidence level was found to be higher for correct responses. The Mann-Whitney test was used to assess the relationship between the obtained answers and the confidence with which GPT-5 provided them.

Confidence distribution analysis revealed a clear distinction; higher confidence levels were associated with correct responses, while lower ratings were more frequent for incorrect answers (Figure 1).

## Discussion

AI is a branch of computer science that focuses on creating devices, systems, and computer software capable of thinking, learning, and making decisions similarly to humans [9]. AI can be used in medicine [10]. Its development in this area has the potential to revolutionize work methods and communication between healthcare professionals and patients. In the medical context, AI is primarily used in diagnostics, personalized healthcare, screening and prevention, administrative automation, research coordination, and medical education [11].

Despite these opportunities, significant challenges remain, including data privacy, cybersecurity risks, accountability for AI-driven decisions, potential complacency among healthcare professionals, and over-reliance on technology [12].

The PES exam in child and adolescent psychiatry is a rigorous test, requiring both theoretical expertise and clinical problem-solving skills. This study explores the efficacy of GPT-5 in the previously mentioned exam. The program achieved an accuracy rate of 80.8%, comfortably exceeding the passing threshold and outperforming earlier versions reported in comparable studies. Such results suggest that modern AI models can achieve performance levels that would allow them to be exempt from the spoken part of the exam. 

The latest GPT-5 model easily reached the required score threshold (60%) and responded consistently to both theoretical and clinical questions, suggesting its superiority in effectiveness to previous versions. Compared with GPT-3.5 and GPT-4.0, the current model demonstrates notable advancements in medical reasoning. 

It is important to emphasize that the observed differences were statistically significant, as confirmed by the Mann-Whitney U test. The consistency of the study’s findings across different assessments underscores the potential of AI as a tool for supporting medical education, while also highlighting the need for careful integration of AI systems and expert oversight of the process. The results may be compared with those reported in a study on the dermatology specialization examination [13]. In that study, the performance of ChatGPT-3.5 and ChatGPT-4.0 was evaluated on 120 questions from the National Specialty exam in dermatology, with ChatGPT-4.0 achieving a minimum score of 70% for the Polish version and a minimum score of 80% for the English version. In both this study and the one mentioned above, the model exceeded the passing threshold of 60%, thereby demonstrating its potential as educational support, even in more demanding specialty examinations.

In medicine, acquiring knowledge and analyzing data are processes that require sufficient time and experience to effectively collaborate with patients. Effective communication with patients and the relationship between them and medical professionals is also crucial. This aspect also appears in questions included in the USMLE test [14]. Brin et al. (2023) examined how ChatGPT can be used to develop skills related to communication, ethics, empathy, and professionalism by analyzing questions from the USMLE and the AMBOSS question database [15]. In their study, GPT-4.0 achieved a better score than previous versions (90.0% compared to 62.5%). This is a crucial aspect, especially in the field of child and adolescent psychiatry, where AI would enable the development of soft skills, which would translate into better patient-physician collaboration. Therefore, it is worth considering conducting similar studies in Poland to assess the potential of AI models in the national medical education system and clinical practice.

The results of our study align with observations from other analyses, including research on the cardiology specialization exam [16]. That study also reported no significant differences in performance between clinical and theoretical questions, while higher confidence declared by the model was more often associated with correct answers. A similar pattern was observed in studies within the field of nuclear medicine [17]. These findings fit into the broader body of research on the use of language models in medicine. Our study demonstrates how AI can operate in a highly specialized pediatric context, providing insights into both diagnostic reasoning and its potential educational utility, while previous research in fields such as cardiology and radiology has reported similar trends in answer accuracy and the level of declared confidence [3,8,13].

This may suggest that the potential of AI in medical education is of a universal nature, although it still requires further validation across different clinical and educational contexts. The technology may occasionally produce answers that appear persuasive yet are inaccurate, which in clinical practice could entail significant risks. Therefore, AI ought to be regarded as a complementary aid in medical education and training, rather than a replacement for the expertise of qualified professionals. It should be emphasized that, despite promising outcomes, AI still presents certain limitations. This may suggest that the potential of AI in medical education is of a universal nature, although it still requires further validation across different clinical and educational contexts. It should be emphasized that, despite promising outcomes, AI still presents certain limitations. The technology may occasionally produce answers that appear persuasive yet are inaccurate, which in clinical practice could entail significant risks.

One minor limitation of this study is that, although all exam questions were originally formulated in Polish, the analytical tool operated primarily in English. Consequently, the exact wording, potential terminological nuances, and the role of context or implied translation were not fully accounted for. This language discrepancy may have introduced translation inaccuracies, potentially affecting the model’s understanding, performance, and the precision and correctness of the obtained responses. Future research could provide further guidance on question formulation and contextualization, thereby enhancing reproducibility and accuracy. Therefore, AI ought to be regarded as a complementary aid in medical education and training, rather than a replacement for the expertise of qualified professionals.

## Conclusions

GPT-5 demonstrated high accuracy in the 2025 National Specialty Examination of Poland in child and adolescent psychiatry, surpassing the required score threshold (60%), which proves that such systems could facilitate learning and exam preparation. Its ability to provide both answers and confidence estimates positions it as a promising educational tool, helping students identify strengths and weaknesses during exam preparation.

Nevertheless, the integration of AI into medical training and education must proceed with caution. Further research is required to validate its effectiveness across specialties and to establish safe and ethical frameworks. It is to remain alert when choosing to trust its answers, even when proclaimed as “fully confident,” and take “low confidence” answers as a signal for further research. Furthermore, implementation of AI in healthcare should undergo the establishment of rules and standards regarding expert oversight of AI-generated texts to ensure that they complement, not replace, critical thinking and expert judgment, particularly in sensitive areas such as child and adolescent psychiatry.

## Appendices

 Appendix A

**Table 3:** Questions and responses of the GPT-5 model in the polish specialized psychiatric examination for children and adolescents

Question	GPT-5 answers	Confidence
1. Acute stress disorder in a child may be characterized by the following symptoms: play involving themes of death; temper tantrums; memory disturbances manifested by inability to recall important details related to the stressful event; feelings of guilt; chronic agitation and irritability. Correct answer: A. all of the above. B. 1,4. C. 1,2,4. D. 4,5. E. only 5.	A	5
2. Indicate the true statements concerning night terrors in children: they are the first symptom indicating the risk of future paranoid schizophrenia (At Risk Mental State, ARMS); they occur as a physiological phenomenon in about 40% of children and are not pathological if not regular and transient with maturation; they are classified as NREM parasomnias and usually occur in the second half of the night; they are NREM parasomnias and usually occur in the first half of the night; they are REM sleep parasomnias and occur in the second half of the night. Correct answer: A. 1,4. B. 1,2,4. C. 2,5. D. 2,4. E. 1,3,5.	D	5
3. Marysia is a 5-year-old girl raised in atypical conditions. The first 3 months of life she spent with her biological family, where she experienced violence and neglect. Then she was placed in a childcare institution for 3 years. Later, she was transferred to a foster family home, living with three other children. Marysia rarely seeks comfort from caregivers in stressful situations, and when comforted, she does not respond positively to closeness. She also shows episodic aggressive behaviors toward caregivers (inappropriate agitation, shouting, physical aggression such as kicking). She does not respond to visiting adults and does not show joy when adults comment on her developmental progress. Autism spectrum disorder was excluded in her mental state examination. These symptoms have persisted for over a year. What is the most probable diagnosis? A. hyperkinetic disorder with attention deficit. B. oppositional defiant disorder. C. Asperger’s syndrome. D. reactive attachment disorder. E. temporal lobe epilepsy.	D	5
4. A 13-year-old boy has been taking aripiprazole for 3 days due to chronic motor and vocal tics. Since starting the medication, he has shown psychomotor agitation that was not present before. He cannot sit still, keeps changing position, often stands up and walks around the classroom. What is the most probable cause of his movement disorder? A. akathisia – an adverse effect after initiating treatment. B. tardive dyskinesia – an adverse effect after initiating treatment. C. muscle dystonia – an adverse effect after initiating treatment. D. parkinsonian tremor – an adverse effect after initiating treatment. E. sudden “rebound effect” indicating a positive response to treatment in the initial phase.	A	5
5. Indicate the correct description of the psychopathological phenomenon of folie à deux (shared psychotic disorder): A. in the induced person, the adopted delusional system evolves. B. in both close individuals, identical delusions develop simultaneously. Both share risk factors and genetic predisposition. After separation, symptoms usually do not resolve. C. a psychotic person imposes their delusional system on a close person. After separation, symptoms in the second person quickly disappear. D. a psychotic person imposes their delusional system on a close person, who initially resists but eventually adopts it. After separation, symptoms do not resolve. E. a healthy person, by observing the functioning of the psychotic person, consciously decides that the delusions are true.	B	5
6. According to epidemiological statistics, boys are several times more likely than girls to die by suicide. The reasons for male predominance do NOT include: A. different social roles. B. different ways of expressing emotions. C. lower readiness to seek help. D. more frequent use of less violent and less lethal suicide methods. E. more frequent use of violent suicide methods.	D	5
7. Neuroanatomical and neurofunctional changes in obsessive-compulsive disorder involve: A. disrupted connections between the cerebellar vermis and the precentral gyrus of the cortex. B. abnormalities in the CA3 region of the hippocampus: increased neuronal death. C. disruptions in cortico-subcortical circuits: striatum, anterior cingulate cortex (ACC), orbitofrontal cortex, and caudate nucleus. D. abnormalities in the insular cortex gyri. E. abnormal signaling between the insular cortex and amygdala.	C	5
8. A 17-year-old patient was admitted to a psychiatric ward with suspected psychotic episode. Mental status exam revealed formal thought disorders (verbosity, loose associations, bizarre connections). He shows decreased psychomotor drive and denies perceptual disturbances. Physical exam: bilateral conjunctival injection. Which test will determine the most probable cause? A. Leiter performance scale test. B. Raven’s Progressive Matrices test. C. CT scan of head and neck. D. urine drug screening (multi-test). E. functional MRI of the brain.	D	5
9. Palilalia is a type of tic: A. complex – repetition of one’s own words. B. complex – repetition of others’ words. C. simple – repetition of others’ words. D. simple – repetition of one’s own words. E. simple motor tic – repetition of movements.	A	5
10. Chromosomal aberrations detectable with karyotype testing may underlie the ASD phenotype in about: A. 0.5% of individuals. B. 1% of individuals. C. 5% of individuals. D. 10% of individuals. E. 50% of individuals.	C	5
11. The classic narcolepsy tetrad does NOT include: A. excessive daytime sleepiness. B. cataplexy (sudden loss of muscle tone). C. hypnagogic hallucinations. D. sleep paralysis. E. catalepsy (rigid posturing).	E	5
12. Drugs primarily used in opioid withdrawal syndrome are: A. clonidine and methylphenidate. B. clonidine and topiramate. C. clonidine and methadone. D. methylphenidate and topiramate. E. methadone and topiramate.	C	5
13. Indicate the false statement about Graves’ disease: A. anxiety disorders occur in up to 60% of patients. B. thyrotoxicosis may present as a manic episode. C. psychotic episodes secondary to thyrotoxicosis are observed. D. goiter is not observed in the course of the disease. E. Hashimoto encephalopathy may occur.	D	5
14. According to DSM-5 diagnostic criteria, post-traumatic stress disorder: A. is diagnosed in a person exposed to death, threatened death, serious injury, or sexual violence. B. is diagnosed in a person exposed to any significant trauma – in adolescents this may include school or homework stress. C. does not occur before age 6. D. cannot present with depersonalization or derealization. E. always develops at least 6 months after the traumatic event.	A	5
15. According to diagnostic criteria, attachment disorder is diagnosed in a child: A. with severe inadequate care involving deprivation of basic needs, neglect of social contact, repeated changes of caregivers, and being raised in atypical conditions. B. with a persistent pattern of emotionally withdrawn behavior toward adults, manifested by lack of seeking comfort in distress and lack of response to comfort. C. with a persistent pattern of emotionally withdrawn behavior toward adults, manifested, e.g., by lack of seeking comfort in distress or lack of response to comfort. D. A and B are true. E. A and C are true.	E	5
16. Body dysmorphic disorder most commonly begins at age: A. 6–7 years. B. 12–13 years. C. 20–25 years. D. after age 30. E. not observed below age 18.	B	5
17. The most common monogenic cause of intellectual disability and autism spectrum disorders is a mutation in the gene: A. FMR1 (Fragile X syndrome). B. MeCP2 (Rett syndrome). C. IKBKG (Bloch-Sulzberger syndrome). D. FBN1 (Marfan syndrome). E. OXTR.	A	5
18. Indicate the true statement about obsessive-compulsive disorder: A. PANDAS syndrome may underlie OCD. B. OCD prevalence is estimated at about 0.35–4%. C. first OCD episode usually occurs around ages 7–8. D. SSRIs are the main pharmacological strategy. E. all of the above.	E	5
19. Indicate the true statement about sleep disturbances in children with autism spectrum disorder (ASD): A. affect about half of children with ASD, usually take the form of insomnia due to poor sleep hygiene, and do not influence ASD core symptoms. B. affect about half of children with ASD, usually result from comorbid psychiatric disorders, but do not exacerbate ASD symptoms. C. affect about 80% of children with ASD, usually insomnia related to poor sleep hygiene, leading to worsening of ASD symptoms. D. affect about 80% of children with ASD, often reported by parents, mainly prolonged sleep onset, but do not exacerbate ASD symptoms. E. affect about 30% of children with ASD, usually due to comorbid psychiatric disorders, and may exacerbate ASD symptoms.	C	5
20. In very severe, treatment-resistant childhood tic disorders, including pharmacotherapy, the following can be considered: A. deep brain stimulation (DBS) or transcranial magnetic stimulation (TMS). B. EEG biofeedback. C. EMDR therapy (Eye Movement Desensitization and Reprocessing). D. electrotherapy (ET). E. supportive treatment with cannabis derivatives (medical marijuana THC).	A	5
21.In the case of no improvement in the functioning of a school-aged child diagnosed with ADHD after treatment with CR-form methylphenidate (as part of comprehensive therapy), one should consider switching to: A. atomoxetine. B. lisdexamfetamine. C. clonidine. D. guanfacine. E. short-acting methylphenidate.	B	5
22. A 15-year-old boy complains of about one month of loss of energy, low mood, and difficulty concentrating. In psychiatric assessment, he presents low self-esteem but denies sleep disturbances, appetite changes, or suicidal thoughts. His symptoms cause slightly worse school performance, although he attends classes regularly. The examining doctor cannot establish a link between the mood change and any significant life event. After excluding somatic causes, as first-line management, the doctor should propose: A. initiation of sertraline and cognitive-behavioral therapy (CBT) or dialectical behavior therapy (DBT). B. initiation of fluoxetine and individual therapy in the CBT or interpersonal therapy model. C. initiation of fluoxetine and individual therapy in the CBT or psychodynamic model. D. initiation of fluoxetine and supportive psychotherapy. E. psychotherapy only (CBT or interpersonal therapy).	E	5
23. What information regarding course and prognosis should the physician provide to the parents of a 6-year-old girl diagnosed with selective mutism (SM): A. SM is a remitting disorder; longer periods of improvement can be achieved only with intensive psychotherapy combined with pharmacotherapy. B. SM most often has a fluctuating course with periods of improvement and exacerbation, but full symptomatic and functional remission is rare. C. SM is a transient disorder, and symptoms often completely resolve during school years, especially with early psychotherapy. D. SM is chronic; prognosis in preschoolers is worse than in older children, although early psycho- and pharmacotherapy can improve it. E. SM is chronic, usually persisting into adulthood, with high comorbidity (especially with other anxiety disorders), but properly conducted psychotherapy can alleviate symptoms and improve functioning.	C	5
24. Features with the highest prognostic value for development of borderline personality disorder are: A. sense of emptiness, fear of abandonment, disturbed sense of identity. B. sense of emptiness, comorbid eating disorders, interpersonal conflicts. C. disturbed sense of identity, neurotic anxiety, ambivalent interpersonal relations. D. fear of abandonment, impulsivity, self-destructive behaviors. E. fear of abandonment, interpersonal conflicts, emotional lability.	D	5
25. A 13-year-old boy has been hyperactive since preschool. Since starting school, teachers have noted significant concentration and attention difficulties. For about 8 months, he has become vulgar and quarrelsome. For over 6 months, he has been associating with a peer group, with whom he broke into a neighbor’s house to obtain money for a trip to the mountains. During the trip, police found him after his family reported him missing the day before. First-line pharmacological treatment, alongside sociotherapeutic interventions, should be: A. risperidone. B. methylphenidate or atomoxetine. C. risperidone combined with methylphenidate. D. valproate derivatives. E. clonidine.	B	5
26. The mother of a 16-year-old boy repeatedly visits the family doctor, demanding multiple tests (and repetition of already done ones) to explain what she calls the “corpse-like pallor” of her son’s skin, and seeks interventions to eliminate it. She also encourages him to use blush on his cheeks. The doctor does not find pallor, physical exam is normal, and additional test results are within normal limits. In the mother, one may suspect: A. Munchausen syndrome by proxy. B. hypochondriacal disorder. C. somatization disorder. D. obsessive-compulsive disorder. E. body dysmorphic disorder by proxy.	E	5
27. The following are not qualitative memory disturbances: A. memory illusions. B. cryptomnesia. C. ekmnesia. D. misidentification delusions. E. confabulations.	D	5
28. Which psychopathological phenomena may explain glossolalia? A. memory hallucinations. B. misidentification delusions. C. cryptomnesia. D. selective amnesia. E. hypermnesia.	C	5
29. Indicate the false statement about a patient presenting confabulations: A. suggestible. B. affectively flat content. C. variable content of confabulations. D. usually features of organic CNS damage. E. confabulation content is intentionally produced.	E	5
30. “It is latent, implicit; one type is procedural memory concerning how to perform certain actions in a specific situational context (‘knowing how’), e.g., remembering how to tie shoes.” The description refers to: A. declarative memory. B. non-declarative memory. C. working memory. D. long-term memory. E. episodic memory.	B	5
31. Indicate the false statement regarding obsessions: A. they are recurrent. B. they are images, fantasies, or impulses. C. they are recognized as one’s own thoughts. D. they are egosyntonic. E. they cause significant subjective distress.	D	5
32. The following is not a thought process disorder: A. loosening of associations. B. thought blocking. C. flight of ideas. D. mutism. E. mantism.	D	5
33. The intellectual functioning level of an adult corresponding to the social maturity of a 10-year-old child can be interpreted approximately as: A. profound intellectual disability. B. severe intellectual disability. C. moderate intellectual disability. D. mild intellectual disability. E. borderline intellectual functioning.	D	5
34. According to Paul Ekman, the following is not a basic (primary) emotion identifiable from facial expression: A. anger. B. fear. C. joy. D. trust. E. disgust.	D	5
35. Under Polish law, a “minor” is a person: A. under 18 years old who has not committed an offense. B. who committed an offense and is under 17 years old. C. who committed a repeat offense between ages 17 and 18. D. who was found guilty of an offense before age 15. E. who was found guilty of an offense before age 21.	B	5
36. In A.E. Kazdin & J.R. Weisz, Evidence-Based Psychotherapies for Children and Adolescents, the Young Autism Project (YAP) includes: discrete trial training (DTT); involvement of students after coursework in behavior analysis; pharmacological treatment of comorbid disorders; case supervisors’ interventions; parental involvement. Correct answer: A. 1,2,3. B. 1,3,5. C. 2,4,5. D. 1,2,3,4. E. 1,2,4,5.	E	5
37. Research indicates a shared genetic basis of Tourette syndrome and: A. generalized anxiety disorder. B. obsessive-compulsive disorder. C. dissociative disorders. D. eating disorders. E. depressive disorders.	B	5
38. If during history-taking the doctor learns that a 16-year-old girl is sexually active with her 14-year-old boyfriend, the doctor is obliged to: A. notify the family court to review the patient’s care situation. B. further explore the age and habits of the patient’s peer group. C. periodically assess the patient’s risk of suicide attempts and self-harm. D. provide psychoeducation on risky behaviors and assess the degree of demoralization. E. provide psychoeducation on contraception and prevention of unwanted pregnancy	A	5
39. If a 16-year-old patient tells the doctor he must kiss the desk and generally has to kiss objects, the doctor may suspect the patient has the following tics: A. projected/phantom tics. B. cognitive/mental tics. C. motor tics. D. sensory tics. E. vocal tics.	C	5
40. In A.E. Kazdin & J.R. Weisz, the “Oregon Multidimensional Treatment Foster Care (MTFC) Program” concerns: A. juvenile offenders placed in correctional institutions. B. parents of children with fetal alcohol syndrome (FAS) lacking parenting skills. C. children with aggressive behaviors and school adaptation difficulties. D. adolescents who committed offenses, with the goal of returning them to their family of origin. E. adolescents with anxiety disorders from broken families exposed to ineffective therapy.	D	5
41. The Big Five theory of P. Costa and R. McCrae concerns: description of Cluster D personality disorders; personality traits; intensity of victimization; confirmed by empirical studies. Correct answer: A. 3,4. B. 2,4. C. 1,2,3. D. 1,3,4. E. 1,2,4.	B	5
42. Behavioral addiction to the Internet is characterized by: A. decreased tolerance for Internet use. B. decrease in externalizing symptoms. C. impaired sensory-motor coordination. D. increased sense of being in the focus of other Internet users. E. increased levels of anxiety and depressive symptoms.	E	5
43. Indicate the true statements: genetic factors account for 76% of ADHD occurrence; fMRI studies in ADHD patients show abnormalities in multiple neural circuits, including hyperactivation of the frontoparietal area, putamen, ventral attention network, as well as hyperactivation of the default mode network and visual circuits; in the latest meta-analysis by Guilherme Polanczyk, ADHD prevalence in the general population of children and adolescents was estimated at 3.4%; core ADHD symptoms are inattention, hyperactivity, impulsivity; diagnosis of ADHD requires symptoms in only one environment in which the child functions. Correct answer: A. 1,2,4. B. 1,3,4. C. 2,3,4. D. 1,3,5. E. all of the above.	B	5
44. The following is not a perceptual disturbance: A. pareidolia. B. illusions. C. hallucinations. D. unrevealed perceptions. E. psychosensory disturbances.	D	5
45. Indicate the true statements: flight of ideas – extreme acceleration of thought processes, speaking many, usually chaotically connected contents (logorrhea); thought blocking – complete halt of a previously slowed thought process (speech); mantism – feeling of burdensome excess of spontaneously emerging threads and topics of thinking, usually disrupting it; expressed in reduced fluency and clarity of speech; tangentiality – repeated responses to questions with answers only slightly related or unrelated to the question; iterations – verbal stereotypies often with motor stereotypies: tapping rhythms unrelated to previous speech. Correct answer: A. 1,3. B. only 1. C. 1,2,3. D. 1,3,4. E. 2,3,4,5.	D	5
46. Severe intellectual disability is characterized by intellectual functioning: A. 1–2 standard deviations below the mean. B. 2–3 standard deviations below the mean. C. 3–4 standard deviations below the mean. D. 4–5 standard deviations below the mean. E. more than 5 standard deviations below the mean	C	5
47. Tools used in diagnosing children and adolescents include: Youth Depression Rating Scale – Revised (YDRS-R); Children’s Yale-Brown Obsessive Compulsive Scale (CY-BOCS); Children’s Manifest Anxiety Scale (CMAS); Spence Children’s Anxiety Scale (SCAS); Child Behavior Checklist (CBCL). Correct answer: A. all of the above. B. none of the above. C. 2,3,4. D. 1,2,5. E. 2,3,4,5.	E	5
48. Four of the 15 most common diseases responsible for years lived with disability (YLDs) are mental health disorders. Indicate them: unipolar depressive disorders; alcohol use disorders; self-injuries; psychoses; Alzheimer’s disease and other dementias. Correct answer: A. 1,2,3,4. B. 1,2,3,5. C. 2,3,4,5. D. 1,3,4,5. E. none of the above.	A	4
49. Indicate the true statements: Level I of the psychiatric care model for children and adolescents includes community psychological-psychotherapeutic care centers; Level II includes outpatient mental health clinics for children and adolescents; Level II includes day psychiatric wards for children and adolescents; Level III includes multidisciplinary inpatient psychiatric care centers; Level III must incorporate Level II. Correct answer: A. 1,2. B. only 3. C. 1,2,3,4. D. 1,2,3. E. all of the above.	E	5
50. Biological factors underlying anxiety disorders include: hypersensitivity of the respiratory center; increased production of noradrenaline metabolites; shortened latency of REM sleep and stage IV slow-wave sleep; increased GABA levels leading to CNS hyperexcitability; functional and structural changes in the frontal and parietal lobes. Correct answer: A. 1,2. B. 1,2,3. C. 1,3,4,5. D. 2,3,5. E. 1,2,3,4.	B	5
51. Indicate the true statement regarding ASD (Autism Spectrum Disorder): A. heritability in ASD—that is, the contribution of genetic factors to disease risk—is among the highest of all psychiatric disorders and is 0.4–0.9. B. in about 30% of patients, the ASD phenotype is due to chromosomal aberrations visible on karyotyping. C. population studies have found, among other things, a significant increase in ASD risk in first-degree relatives and a high concordance rate in dizygotic twins of 60–90%. D. a sign of delayed speech development in children with ASD is the absence of simple (two-word) sentences before the age of 1 year. E. maternal obesity, diabetes, and hypertension prior to conception are protective factors against ASD in the child.	A	5
52. The effect size for psychostimulant medications in ADHD (Attention Deficit Hyperactivity Disorder) therapy reaches: A. 0.1. B. 0.3. C. 0.4. D. 1.0. E. 1.2.	E	5
53. The most common adverse effects of psychostimulants are: loss of appetite; rash; abdominal pain; headaches; bradycardia. Correct answer: A. 1,2. B. 1,2,3. C. 1,3,4. D. 4,5. E. only 5.	C	5
54. A tool helpful in diagnosing ASD is: A. Raven’s Matrices Test. B. DSR. C. SB5. D. ASRS. E. STATIC.	D	5
55. According to DSM-5 diagnostic criteria, a hypomanic episode may be suggested by the following set of symptoms: the patient has a decreased need for sleep; the patient has inflated self-esteem; symptoms last until the seventh day; psychotic features are present; symptoms are caused by the physiological effects of psychoactive substances. Correct answer: A. 1,2,5. B. 2,3,5. C. 1,3,5. D. 1,2,3. E. 2,4,5.	D	4
56. Indicate the true statement: A. engaging in goal-directed activities increases tics. B. a constant movement pattern over months/years is characteristic of tics. C. a constant localization of movement over months/years is characteristic of stereotypies. D. the subjective sensation associated with tics is usually perceived as pleasant and desirable. E. sleep is a factor that exacerbates compulsions.	C	5
57. Compared to the typical clinical picture, the symptoms that help distinguish ADHD from a manic episode in bipolar disorder are: grandiosity is characteristic of ADHD and uncharacteristic of mania; elevated mood is characteristic of mania and uncharacteristic of ADHD; decreased need for sleep is characteristic of ADHD and uncharacteristic of mania; impulsivity occurs in ADHD and does not occur in mania; increased sexual drive is characteristic of mania and uncharacteristic of ADHD. Correct answer: A. 1,3,4. B. 1,4. C. 2,5. D. 2,4,5. E. 1,3.	C	5
58. According to the Polish Act of 9 June 2022 on Support and Resocialization of Juveniles (Journal of Laws 2022, item 1700, consolidated text), the following is not included among educational (rehabilitative) measures: A. referral to a probation center. B. a ban on driving any vehicles or vehicles of a specified type. C. supervision by the juvenile’s parents or guardian. D. placement in a youth sociotherapy center. E. placement in a youth educational center.	B	5
59. According to the DC:0–5 diagnostic classification, a child’s developmental competence in the emotional, social–relational, language–social communication, cognitive, motor, and physical domains should be assessed using axis: A. I. B. II. C. III. D. IV. E. V.	E	5
60. The following are not among medications registered in Poland for selected indications in persons under 18 years of age: A. ziprasidone. B. lithium. C. amisulpride. D. sulpiride. E. chlorpromazine.	A	5
61. Indicate the false statement regarding the diagnostic criteria for adjustment disorders in DSM-5: A. onset of emotional or behavioral symptoms in response to an identifiable stressor within 4 weeks of its occurrence. B. symptoms are not manifestations of bereavement. C. symptoms do not persist longer than 6 months after the stressor or its consequences have ceased. D. the stress-related disturbance does not meet criteria for another mental disorder and is not merely an exacerbation of a pre-existing mental disorder. E. none of the above.	A	5
62. Which of the clinical disorders is not included in the DC:0–5 classification? A. early childhood schizophrenia. B. developmental coordination disorder. C. excessive crying. D. early childhood depression. E. sensory overreactivity.	B	5
63. Which diagnostic tool is not used in the assessment of autism spectrum disorder? A. M-CHAT-R/F. B. STAT. C. ASAS. D. CAST. E. all of the above are used in ASD diagnostics.	C	5
64. Indicate the false statement about Fragile X syndrome: A. it is a common cause of intellectual disability. B. a characteristic phenotypic feature in boys is small head circumference and small testes during puberty. C. it is most often associated with moderate to severe intellectual disability. D. in early childhood, patients may present with hyperactivity, motor stereotypies, and impaired eye contact. E. about 30% of men with this diagnosis present with autism spectrum symptoms.	B	5
65. The feeling of a burdensome excess of spontaneously emerging threads and topics of thought is called: A. metonymy. B. verbigeration. C. mantism. D. mutism. E. perseveration.	C	5
66. Williams syndrome is: A. an example of a chromosomal aberration caused by a deletion on the short arm of chromosome 7. B. a disorder in which children show excessive ease in establishing social contact. C. a disorder that does not significantly affect the cognitive development of affected children. D. a syndrome that occurs more often in boys. E. a syndrome belonging to dynamic mutations.	B	5
67. A disorder characterized by excessive ease in establishing social relations: A. develops on a basis other than reactive attachment disorder. B. is an expression of trust shown by a child raised in a friendly environment. C. usually occurs in children under 9 months of age. D. involves actively approaching and making contact with unfamiliar adults, excessively friendly behavior, and communication. E. none of the above.	D	5
68. A child victim of abuse, admitted urgently to a psychiatric hospital due to suicidal thoughts and intentions reported in the care institution where they have been staying for about 2 months, complains of intrusive memories, inability to recall entire periods of childhood at home, memory problems, concentration difficulties, and sudden vivid flashbacks of past abuse. The physician suspects post-traumatic stress disorder. Which additional symptoms may confirm this suspicion? A. sleep disturbances in the form of nightmares. B. repetitive play reenacting traumatic events experienced. C. avoidance of things, people, or events related to the trauma. D. hyperarousal, problematic behaviors. E. all of the above.	E	5
69. A 12-year-old child is referred to a psychiatrist for increasing anxiety, accompanied by panic attacks with palpitations, dyspnea, excessive sweating, pallor, headaches, and hypertension. These symptoms have been accompanied by low mood and reduced activity. The doctor should: A. diagnose mixed anxiety–depressive disorder and initiate pharmacotherapy. B. diagnose panic disorder, recommend psychotherapy, and if ineffective, introduce pharmacotherapy. C. conduct differential diagnosis, exclude somatic causes such as pheochromocytoma, and only then initiate symptomatic treatment. D. conduct a thorough clinical, individual, and family history, then refer for psychological diagnostics, psychoeducation, and psychotherapy. E. conduct a thorough clinical and environmental history, then implement psychoeducation involving the patient and family, followed by desensitization training.	C	5
70. Secondary endocrine psychopathological syndromes include: A. severe suicidal tendencies. B. significant mood lability. C. aggressive behaviors. D. acute psychotic decompensation. E. all of the above.	E	5
71. A 5-year-old child with normal prior development and speech is brought to a specialist due to increasing communication difficulties: the child seems not to understand parental speech, begins speaking in simpler sentences, speaks spontaneously less often, and speech is less fluent. EEG shows paroxysmal activity. The physician suspects: A. Rett syndrome. B. Landau-Kleffner syndrome. C. selective mutism. D. childhood-onset fluency disorder (stuttering). E. none of the above.	B	5
72. A 4-year-old child in foster care, verbally communicative, performing educational tasks at peer level, but with difficulties in peer cooperation: tends to isolate, is unresponsive to encouragement, rarely reciprocates communication. The physician suspects: A. reactive disorders. B. autism spectrum disorder. C. intellectual disability. D. social skills deficit. E. none of the above.	B	4
73. Indications for MRI of the brain and spinal cord do not include: A. suspected neoplastic process. B. suspected neurodevelopmental disorders. C. suspected neurodegenerative processes. D. follow-up after neurosurgical treatment with metallic clips. E. follow-up in patients with certified cochlear implants.	D	5
74. In studies involving children and adolescents, obtaining informed consent for voluntary participation is: A. not necessary. B. necessary, based on the principle of respect for persons in human research. C. necessary, based on the principle of beneficence in human research. D. necessary, based on the principle of justice in human research. E. none of the above.	B	5
75. Trichotillomania in young children: A. often begins as an automatic gesture associated with thumb-sucking, pacifier use, bottle-feeding, watching TV, or falling asleep. B. may be associated with anxious, tense, and anger-filled family relationships. C. in preschool age affects boys and girls equally in terms of pleasure from hair-pulling. D. A and B are true. E. A, B, and C are true.	E	5
76. Parents bring their 12-year-old daughter to a psychiatrist one week after discharge from a psychiatric hospital. Before admission she had no psychiatric or psychotherapeutic treatment. She was admitted due to a suicide attempt, but parents sought early discharge. They now want “higher standards of care” than in the hospital, expecting more active treatment. The girl is affectively flat, with limited facial expression, answers after delay, and reports voices commanding her to kill herself. She says she does not want to harm herself but cannot guarantee safety. The doctor should: A. refer her again to hospital due to high suicide risk. B. provide care without referral to other specialists. C. provide care while suggesting family therapy. D. provide care while suggesting individual child therapy. E. recommend individual and family therapy without medical care.	A	5
77. A 13-year-old boy, recently placed in an orphanage, presents to the psychiatric ER with his legal guardian. He has a history of repeated psychiatric hospitalizations for suicidal thoughts related to domestic abuse. Since admission to the orphanage, he has been verbally aggressive toward staff and peers, once self-harmed with a razor, and refuses school. The guardian fears for his life. On exam, the boy denies suicidal thoughts, mood and affect are adequate. He loudly expresses dissatisfaction with his situation and openly requests hospital admission, recalling prior positive experiences. In this case: A. he should be admitted unconditionally. B. he should be admitted due to previous positive hospital experiences. C. he should be admitted due to the self-harm incident. D. he should be admitted due to past suicidal problems. E. there are no grounds for acute admission; instead, outpatient care should be arranged (e.g., level I or II center).	E	5
78. The validity of a psychiatric hospital referral issued by a physician expires after: A. 7 days. B. 12 days. C. 13 days. D. 14 days. E. 21 days.	D	5
79. Among educational measures to prevent juvenile delinquency and demoralization, the following is notincluded: A. admonition. B. obligation to repair damage. C. probation officer supervision. D. driving ban. E. placement in a treatment facility.	D	5
80. Which of the following symptoms is not typical for Overactivity Disorder of Toddlerhood? A. repeatedly asking parents questions to obtain reassurance. B. talking excessively. C. frequently climbing on furniture or inappropriate objects. D. difficulty waiting for one’s turn in conversation. E. A and B are true.	A	5
81. The differential diagnosis of disorders of selective attachment in childhood includes: A. pervasive developmental disorders, including Asperger’s syndrome. B. organic brain damage. C. hospitalism in children. D. A and B are true. E. A, B, and C are true.	E	5
82. Epilepsy treatment with a ketogenic diet, if satisfactorily effective, should last: A. at least 2 years. B. no longer than 2 years. C. no longer than 6 months. D. one month after clinical improvement is sufficient. E. once satisfactory seizure control is achieved, the diet should be discontinued.	A	4
83. Among the symptoms of increased intracranial pressure in older children, the following are not characteristic: A. headache. B. vomiting. C. papilledema. D. diplopia. E. scalp numbness.	E	5
84. In diagnosing narcolepsy, the following tests should be performed: A. several days of inpatient observation to monitor nap frequency and duration. B. multiple sleep latency test (MSLT) preceded by overnight polysomnography. C. 24-hour EEG recording. D. 24-hour video-EEG. E. no additional tests are necessary; history alone is decisive.	B	5
85. Simple febrile seizures do not require prophylactic treatment. However, for acute home management when a seizure occurs, the following should be used: A. rectal phenobarbital. B. rectal diazepam enema or buccal midazolam. C. oral diazepam and buccal midazolam. D. only antipyretics should be given. E. no medications should be used at home.	B	5
86. Alice in Wonderland syndrome—with paroxysmal symptoms of distortions in body shape and size (metamorphopsias), distance, position, and size of objects—is in pediatric neurology a description of: A. migraine aura in children. B. epileptic aura. C. epileptic seizures with temporal symptomatology. D. so-called episodic syndromes occurring only in children and precursors of migraine. E. such a symptom complex has not been described to date.	A	5
87. The Glasgow scale assesses: A. eye opening, pupil status, presence of limb paresis. B. eye opening, motor response, verbal response. C. verbal response, presence of headache, pupil status. D. verbal response, pupil status, presence of limb paresis. E. pupil status, eye opening, verbal response.	B	5
88. Dysarthria is a speech disorder caused by damage to the following anatomical structures: A. developmental defects of the larynx and vocal folds. B. cortical speech centers. C. centers or pathways innervating the speech organs. D. injury to the tongue and lips. E. none of the above.	C	5
89. The following is not a dissociative syndrome: A. fugue. B. trance. C. multiple personality (dissociative identity). D. somatization. E. possession.	D	5
90. A young female inpatient with acute psychosis treated with risperidone 8 mg/day develops altered consciousness, marked motor slowing, trismus, increased muscle tone, sialorrhea. Temperature 38.4°C, HR 120/min. Which tests should be done first? A. ALT, AST, GGT. B. CPK, leukocyte count. C. D-dimers, troponin. D. cerebrospinal fluid examination. E. brain MRI.	B	5
91. A 17-year-old boy raised strictly in a Catholic family had sexual intercourse with a woman 10 years older, who was disappointed with his clumsiness. He has been in therapy for a year for mild depressive states and takes sertraline 50 mg/day. He confides to the therapist about premature ejaculation. Indicate the most probable cause: A. current pharmacotherapy. B. anxiety related to the failed relationship and family environment. C. anatomical penile abnormalities. D. urethritis. E. undiagnosed hormonal disorders.	B	5
92. A person with severe intellectual disability can be placed under full guardianship after the age of: A. 13. B. 16. C. 18. D. 21. E. regardless of age.	C	5
93. An 8-year-old child is diagnosed with ADHD. The first-line management includes: A. education of parents and teachers. B. initiation of cognitive-behavioral psychotherapy. C. initiation of family therapy, social skills training, and training aimed at improving academic skills. D. initiation of pharmacotherapy with methylphenidate or atomoxetine. E. A, B, and C are true.	E	5
94. In psychological assessment of children, both rating scales and questionnaires are used to measure: A. psychopathological symptoms and global developmental characteristics of the child. B. medical indicators describing the child’s somatic condition. C. disturbances in the child’s social personality structure. D. indicators of disturbances in the child’s emotional personality structure. E. none of the above.	A	5
95. In a child’s psychological assessment during treatment, it is important to include diagnostic evaluation of: A. critical periods in the child’s development. B. the child’s personal resources. C. social–environmental resources. D. psychosocial risk factors underlying developmental dysfunction. E. all of the above.	E	5
96. Among developmental fears typical after 9 years of age, the most common is fear of: A. darkness. B. injury and physical harm. C. social evaluation. D. animals, supernatural characters from stories and films. E. being alone.	C	5
97. Among the reflexes typical only for the newborn is the reflex of: A. swallowing. B. sucking. C. automatic stepping (walking). D. pupillary light response. E. none of the above.	C	5
98. The Stanford–Binet Intelligence Scales, Fifth Edition (SB5) is one of two tools recommended by the National Consultant for Clinical Psychology to measure intelligence in individuals aged: A. 2–18 years. B. 2–69 years. C. 3–70 years. D. 6–18 years. E. 6–69 years.	B	5
99. Ego defense mechanisms occur in healthy individuals (drive gratification in a civilized, sublimated form, leaving plenty of psychic energy for exploring the world). In this case we speak of healthy or mature defenses, which include: conscious actions allowing us to cope with unpleasant feelings; unconscious actions allowing us to cope with unpleasant feelings; altruism, anticipation, asceticism, humor, identification, sublimation; anticipation, asceticism, humor, displacement, sublimation; altruism, anticipation, asceticism, humor, sublimation. Correct answer: A. 1,3. B. 2,3. C. 2,4. D. 2,5. E. 1,4.	D	5
100. The prefrontal cortex includes the orbitofrontal, dorsoventral, ventromedial regions, and the anterior cingulate gyrus. It is responsible for multiple domains of human functioning. Indicate the true statement regarding a selected prefrontal region’s function: A. the orbitofrontal cortex is responsible for emotion perception, assigning emotional meaning, and emotion control. B. the dorsoventral prefrontal cortex is responsible for planning and goal setting, working memory, abstract thinking, organizing information, and selecting appropriate information from long-term memory. C. the ventromedial cortex is responsible for behavioral control, critical judgment, empathy, and adherence to social norms. D. the anterior cingulate gyrus provides functional integration of all the above prefrontal functions. E. the anterior cingulate gyrus is responsible for planning and goal setting, working memory, abstract thinking, organizing information, and selecting appropriate information from long-term memory.	A	4
101. A landmark event in 20th-century psychiatry was the introduction of new-generation antidepressants. The first new-generation antidepressant was: A. sertraline, introduced in 1987. B. trazodone, introduced in 1990. C. amitriptyline, introduced in 1980. D. escitalopram, introduced in 1990. E. fluoxetine, introduced in 1987.	E	5
102.Under Article 50(2) of the Polish Mental Health Protection Act (u.o.z.p.), a waiver of psychiatric confidentiality is possible in the following cases: sharing confidential information with the physician providing care to the person with mental disorders; providing information to competent government or local administration authorities regarding circumstances whose disclosure is necessary for carrying out social assistance tasks; providing data to the Internal Security Agency, Military Counterintelligence Service, Central Anti-Corruption Bureau, Police, Military Gendarmerie, Border Guard, Prison Service, Government Protection Bureau, and their authorized officers or soldiers to the extent necessary to conduct a security clearance under the classified information protection regulations; providing information to any police officer conducting operational–recognition activities in the scope of searching for and identifying persons; providing confidential information to any physician. Correct answer: A. 1,2,5. B. 2,3,4. C. 1,2,3. D. 1,2,4. E. 3,4.	C	5
103. Differential diagnosis of disorders related to psychoactive substance (PAS) use is difficult and must be considered for the specific diagnostic category and substance. Intoxication with a specific substance should be differentiated from, among others: A. carbon monoxide poisoning, drug intoxication, acute hypoglycemia, hyperglycemia, alkalosis, electrolyte disturbances, acute hepatitis, brain tumor. B. carbon monoxide poisoning, drug intoxication, acute hypoglycemia, hyperglycemia, suicidal behavior, meningitis. C. other PAS intoxication, drug intoxication, acute hypoglycemia, hyperglycemia, diabetic ketoacidosis, metabolic acidosis, acute pancreatitis, meningitis, stroke. D. other PAS intoxication, drug intoxication, alkalosis, electrolyte disturbances, acute pancreatitis, stroke, brain tumor. E. other PAS intoxication, carbon monoxide poisoning, acute pancreatitis, acute peritonitis, diabetic ketoacidosis, metabolic acidosis.	C	5
104. When assessing executive functions in children and adolescents, one considers indications as well as contraindications. Which problem/disorder may be a contraindication to this assessment? A. psychosis. B. learning difficulties. C. suspected ADHD. D. mood disorders. E. autism spectrum disorders.	A	4
105. Indicate the true statements about speech development in children: a child responds to their own name around the 8th month of life; the rate of speech development in children depends on sex; babbling appears between 6 and 8 months; between 18 and 24 months, developmental echolalia may be observed; a child usually understands and follows simple commands after the 14th month of life. Correct answer: A. 1,2,5. B. 3,5. C. 4,5. D. 1,3,4. E. 2,4.	D	5
106. Indicate for which cognitive competence the preschool period is a critical period: A. attention. B. perception. C. executive functions. D. thinking. E. memory.	C	5
107. Indicate the false statement regarding psychoeducation of parents of a child with somatic symptom presentations: A. it is necessary to discuss the nature of symptoms. B. it is very important to emphasize the reality of the child’s symptoms. C. with regard to the child’s symptoms, discussing a general model of the disorder is sufficient; individualization is not necessary. D. the nature of trigger factors in the given child should be explained. E. it is important to emphasize the child’s suffering caused by the symptoms.	C	5
108. ADOS-2 is the gold-standard observational assessment in autism diagnostics. It evaluates communication, social interaction, play, creative use of materials, and restricted/repetitive behaviors. Indicate the false statement about ADOS-2: A. it is a standalone diagnostic tool; its result determines the diagnosis. B. it consists of five modules. C. it is used to diagnose children above 15 months of mental age. D. it is used to diagnose adults. E. training is required to obtain authorization to administer it.	A	5
109. A 13-year-old boy was admitted to a psychiatric ward after a suicide attempt. After 2 days, on Saturday evening, his parents withdrew consent for hospitalization due to “bad food” and poor conditions. During discharge evaluation, the boy reveals suicidal thoughts, stating that because of his mother’s intrusive attitude he no longer wants to live and will kill himself after returning home. The on-call physician should: A. discharge the patient to his mother’s care, as per her wish. B. improve ward conditions, e.g., order pizza. C. immediately notify the head of department and wait for their arrival. D. keep the patient without parental consent under Article 28 of the Mental Health Protection Act. E. keep the patient without parental consent under Article 24 of the Mental Health Protection Act.	D	5
110. The target daily dose of clonidine in children for ADHD should be divided into 3–4 doses and is: A. 1–3 (maximum 7) μg/kg body weight/24 h. B. 3–6 (maximum 10) μg/kg bw/24 h. C. 6–9 (maximum 13) μg/kg bw/24 h. D. 9–12 (maximum 16) μg/kg bw/24 h. E. none of the above.	A	5
111. ICD-11 diagnostic criteria for a depressive episode include: a duration of at least 2 weeks of depressive symptoms; two core symptoms of depression: depressed mood or psychomotor retardation; three core symptoms: depressed mood, loss of interest/pleasure, decreased energy or increased fatigability; numerous accompanying symptoms, including psychomotor retardation, decreased energy, and increased fatigability; inner tension/anxiety as an accompanying symptom. Correct answer: A. 1,3. B. 1,2,4. C. 2,4,5. D. 1,3,5. E. 1,3,4,5.	A	5
112. Off-label use of a drug is not considered a therapeutic experiment if: the drug is regarded as a tried-and-tested treatment method; the treatment is recommended by recognized standards and guidelines of other European countries; the treatment has been approved by U.S. government agencies; the physician’s clinical practice indicates it is effective. Correct answer: A. 1,2,3. B. 2,3. C. 1,2,4. D. 1,3,4. E. 2,3,4.	A	5
113. ICD-10 criteria for a moderate depressive episode are met if: at least 2 core symptoms and enough additional symptoms are present so that the total number is at least 6; the person functions in most life areas in a very limited manner; at least 2 core symptoms and at least 2 additional symptoms are present, with a total of at least 4; suicide risk is not judged by the assessor to be very high. Correct answer: A. 1,2. B. 1,2,4. C. 3,4. D. only 1. E. only 2.	C	5
114. The pharmacokinetics of psychotropic drugs differ significantly in children and adolescents compared with adults. Characteristic features in children include: higher total body water and lower fat content; higher plasma albumin concentration; smaller volume of distribution than in adults; faster hepatic metabolism and the need for higher per-kg doses than in adults; the need for more frequent dosing. Correct answer: A. 1,2,4,5. B. 3,4,5. C. 1,3,4. D. 1,5. E. 1,4,5.	E	5
115. Combined treatment with fluoxetine and quetiapine may lead to: A. decreased quetiapine levels, reducing its effectiveness. B. decreased fluoxetine levels, reducing its effectiveness. C. increased quetiapine levels, raising adverse-effect risk. D. increased levels of both drugs. E. no impact on drug levels.	C	5
116. For treating criterion ASD symptoms, NICE recommends: A. psychosocial interventions with proven efficacy. B. antipsychotics and antidepressants. C. gluten-free elimination diet. D. casein-free elimination diet. E. secretin.	A	5
117. DSM-5 diagnostic criteria for ADHD include: six or more symptoms from the “inattention” cluster in children/adolescents for at least 6 months; at least four “inattention” symptoms from age 17 and in adults; six or more symptoms from the “hyperactivity/impulsivity” cluster for at least 6 months; age criterion for onset of ADHD symptoms is 7 years. Correct answer: A. 2,3,4. B. 1,2,3. C. 1,3,4. D. 1,3. E. only 1.	C	5
118. The following is not a consequence of left-hemisphere brain damage: A. catastrophic reaction. B. anosodiaphoria. C. apraxia. D. deficits in learning and recalling verbal material. E. speech disorder.	B	5
119. Psychiatric symptoms resulting from bilateral temporal lobe damage are known as the: A. Capgras syndrome. B. Cotard syndrome. C. Klüver–Bucy syndrome. D. Othello syndrome. E. Ganser syndrome.	C	5
120. An EEG tracing showing low-voltage mixed activity with predominant theta waves and the presence of K-complexes is characteristic of: A. wakefulness with eyes closed. B. wakefulness with eyes open. C. REM sleep. D. stage 3 NREM sleep. E. stage 2 NREM sleep.	E	5
